# The Activation of GABA_A_R Alleviated Cerebral Ischemic Injury via the Suppression of Oxidative Stress, Autophagy, and Apoptosis Pathways

**DOI:** 10.3390/antiox13020194

**Published:** 2024-02-03

**Authors:** Jing Lan, Jiaqi Wang, Shujing Wang, Jia Wang, Sijuan Huang, Yazhou Wang, Yunfei Ma

**Affiliations:** 1 National Key Laboratory of Veterinary Public Health and Safety, College of Veterinary Medicine, China Agricultural University, Beijing 100193, China; 2 Department of Neurobiology, School of Basic Medicine, The Fourth Military Medical University, Xi’an 710032, China

**Keywords:** cerebral ischemia, GABA_A_R, oxidative stress, autophagy, apoptosis

## Abstract

Ischemic stroke is a devastating disease leading to neurologic impairment. Compounding the issue is the very limited array of available interventions. The activation of a γ-aminobutyric acid (GABA) type A receptor (GABA_A_R) has been reported to produce neuroprotective properties during cerebral ischemia, but its mechanism of action is not yet fully understood. Here, in a rat model of photochemically induced cerebral ischemia, we found that muscimol, a GABA_A_R agonist, modulated GABAergic signaling, ameliorated anxiety-like behaviors, and attenuated neuronal damage in rats suffering cerebral ischemia. Moreover, GABA_A_R activation improved brain antioxidant levels, reducing the accumulation of oxidative products, which was closely associated with the NO/NOS pathway. Notably, the inhibition of autophagy markedly relieved the neuronal insult caused by cerebral ischemia. We further established an oxygen–glucose deprivation (OGD)-induced PC12 cell injury model. Both in vivo and in vitro experiments demonstrated that GABA_A_R activation obviously suppressed autophagy by regulating the AMPK-mTOR pathway. Additionally, GABA_A_R activation inhibited apoptosis through inhibiting the Bax/Bcl-2 pathway. These data suggest that GABA_A_R activation exerts neuroprotective effects during cerebral ischemia through improving oxidative stress and inhibiting autophagy and apoptosis. Our findings indicate that GABA_A_R serves as a target for treating cerebral ischemia and highlight the GABA_A_R-mediated autophagy signaling pathway.

## 1. Introduction

Ischemic stroke remains the leading cause of death and disability worldwide, accounting for more than 85% of stroke cases [1]. The phrase ‘ischemic stroke’ refers to blood supply disorders that induce cerebral ischemia and hypoxia, ultimately leading to ischemic damage and necrosis of brain tissue. Early recognition and treatment of cerebral ischemia are critical for improving outcomes and reducing the risk of long-term complications. Therefore, developing more effective targeted drugs is essential to reducing the mortality and morbidity of ischemic stroke. Completing this task will require a deeper understanding of pathogenic mechanisms following ischemic stroke.

The pathology of ischemic brain injury depends on extremely complex pathological processes involving multiple cytotoxic factors and inflammatory cells within the central nervous system (CNS) and the peripheral circulation system [2]. Substantial data have demonstrated the combined effects of oxidative stress, neuroinflammation, and autophagy dysfunction, damaging cells after cerebral ischemia. Oxidative stress, triggered primarily by reactive oxygen species (ROS) and reactive nitrogen species (RNS) in cells and organisms, is considered a major factor in cerebral ischemic injury [3]. The overproduction of ROS and RNS during cerebral ischemia overwhelms the endogenous scavenging capacity of cellular antioxidant defenses and stimulates the peroxidation of lipids, proteins, and nucleic acids, leading to DNA damage and mitochondrial dysfunction, and ultimately to cytotoxicity and cell death [4,5,6]. The CNS is particularly vulnerable to ROS toxicity due to its inherent high oxidative metabolism and low antioxidant enzyme levels [7]. Recently, a variety of studies have confirmed the involvement of nitric oxide (NO)/nitric oxide synthase (NOS) in the process of oxidative stress in diverse disease conditions, such as cerebral ischemia, cardiovascular diseases, and neurodegenerative diseases [8,9]. Notably, the accumulation of ROS leads to the induction of autophagy in cerebral ischemic injury, in which the transcriptional regulatory mechanism of ROS autophagy is considered the primary pathway [10,11].

Autophagy is a precisely regulated biological process dependent on lysosome degradation systems in eukaryotic cells, where it serves as a response to adverse stimuli and facilitates the elimination of misfolded proteins and injured organelles to maintain cellular function and homeostasis [12]. Previous studies have illustrated that autophagy plays a crucial role in the pathophysiological mechanisms of ischemic stroke [13], and that the activation of autophagy promotes the removal and reuse of cytoplasmic constituents, thereby protecting neurons against cell death induced by focal cerebral ischemia [14,15]. However, it has also been suggested that autophagy plays a deleterious role during cerebral ischemic injury. Excessive autophagy, which seems to occur more often in the late stages of the disease, promotes cell membrane and organelle disruption and accelerates neuronal death during cerebral ischemia [16,17]. Obviously, autophagy plays a dual role during cerebral ischemia, which is probably largely dependent on the stage of disease progression [18]. Moreover, researchers have found similar dual roles of autophagy during traumatic brain injury and tumor formation [19,20]. Additionally, during cerebral ischemia, autophagy and multiple other cellular biological processes interact with one another to collectively regulate neuronal cell death or survival. Autophagy, as well as apoptosis or necrosis, are thought to be the critical mechanisms of neuronal injury. Numerous studies have confirmed that Beclin-1 acts as a crossroads, leading to cellular autophagy or apoptosis in ischemic stroke, silencing or inhibiting neuronal apoptosis reduced by Beclin-1 expression, and protecting neurons against damage in oxygen–glucose deprivation (OGD) or ischemic conditions [21,22,23].

The pathophysiology of cerebral ischemia involves cellular excitotoxicity, oxidative stress, autophagy, and cell death processes, forming a complex network of pathophysiological mechanisms. The drugs targeting these signaling mechanisms could possibly serve as therapeutic approaches against ischemic stroke. Research has shown that glucose and oxygen deficiencies during cerebral ischemia induce neuronal cell depolarization and glutamate release, which might contribute to excitotoxicity in neurons [24,25]. The excitotoxic process probably depends on a balance between excitatory and inhibitory mechanisms [26]. γ-aminobutyric acid (GABA) is a major inhibitory neurotransmitter in the central nervous system, and its actions are mediated by at least three different receptor classes, in which the GABA type A receptor (GABA_A_R) is one of the principal GABA receptors [27,28]. The presence of normal GABA levels in the brain during stroke is essential for maintaining neurological function. In addition, GABA signaling disorders caused by cerebral ischemia are also closely related to neurological damage [27]. Recent evidence has suggested that enhancing GABAergic function could decrease glutamatergic activity and confer neuroprotective effects [28]. In view of the importance of GABA in cerebral ischemic injury and the difficulty of GABA crossing the blood–brain barrier, a number of drugs targeting for GABA_A_R activation have been used to attenuate ischemic stroke in clinical and animal tests [27]. Muscimol, a psychoactive isoxazole from Amanita muscaria and related mushrooms, has depressant, sedative-hypnotic, and hallucinogenic characteristics. Upon enteral or parenteral administration, muscimol rapidly crosses the blood–brain barrier, displays selective and potent agonist activity at the GABA_A_R sites, and has been shown to be a highly selective agonist at the ionotropic receptor for the inhibitory neurotransmitter GABA [29,30]. It has been shown that muscimol attenuates neuronal death caused by global or focal cerebral ischemia and ischemia/reperfusion and has neuroprotective effects against brain injury [31,32,33]. Moreover, the activation of GABA_A_R by muscimol can lower NMDAR-induced neurotoxicity in primary cell cultures and limit intense exercise-induced synapse impairment, excessive apoptosis, and hippocampus dysfunction [34,35]. However, the underlying mechanisms of the neuroprotective effects of GABA_A_R activation have not yet been fully elucidated. The current accumulating corpus of works on the protective effects of activated GABA_A_R against cerebral ischemic injury have focused on the inhibitory effects of GABA signaling on excitatory glutamate signaling, whereas studies on the effects of activated GABA_A_R on autophagy are limited. Considering the dual role of autophagy in cerebral ischemia, it is necessary to explore whether GABA_A_R activation exhibiting neuroprotection is closely related to the regulation of autophagy.

In the present study, we used rats to establish a photochemically induced cerebral ischemia model in vivo and an OGD-induced PC12 cell injury model in vitro. These were combined with the application of GABA_A_R agonist to investigate the protective effects of GABA_A_R activation against cerebral ischemia. We then explored the molecular mechanism underlying GABA_A_R neuroprotection related to oxidative stress, autophagy, and apoptosis pathways during cerebral ischemia.

## 2. Materials and Methods

### 2.1. Animals

Male Sprague Dawley rats (8 weeks old) with an average weight of 200 g were purchased from Xinglong experimental animal farm (Beijing, China). The rats were housed in a standardized environment with a humidity of 60 ± 5% and maintained at a controlled temperature of 21 ± 3 °C with a 12 h light–dark cycle. All experimental protocols were approved by the Committee for the Care and Use of Experimental Animals, China Agricultural University (AW92602202-2-1). Every effort was made to minimize suffering and limit the number of animals used.

### 2.2. Cerebral Ischemia Model and Experimental Design

We used a brain ischemia model produced using photochemistry [36,37]. Briefly, the rats were anesthetized with inhaled isoflurane (ZS Dichuang Science Technology Development Co., Ltd., Beijing, China), and anesthesia was maintained via the inhalation of isoflurane through a nose cone. Then, the rats were slowly injected through a tail vein with Rose Bengal dye (50 mg/kg body weight). After making a small incision on the scalp, a craniotomic window was cut over the motor cortex, with the center located at a coordinate of 2 mm posterior to the bregma and 2 mm lateral to the midline. Focal cerebral ischemia was induced using irradiation with a halogen cold light source (color temperature 3200 K) on the skull for 25 min.

Experiment 1: Twenty-four rats were randomly assigned to four groups: the control group (Con), the sham-operated group (Sham), the cerebral ischemia model group (Is), and the muscimol group (Mus). The rats were intraperitoneally injected with muscimol (1.5 mg/kg body weight) [38] at 30 min before cerebral ischemia. As a control, the same amount of saline was administrated to rats of other groups.

Experiment 2: Thirty rats were randomly assigned to five groups: the control group (Con), the sham-operated group (Sham), the cerebral ischemia model group (Is), the muscimol group (Mus), and the L-NAME group (L-NAME). In the Mus group, the rats were intraperitoneally injected with muscimol (1.5 mg/kg body weight) at 30 min before cerebral ischemia. The rats in the L-NAME group were intraperitoneally injected with 1% L-NAME (10 mg/kg body weight) for 20 min prior to cerebral ischemia. As a control, the same amount of saline was administrated to rats of other groups.

Experiment 3: Twenty-four rats were randomly assigned to four groups: the sham-operated group (Sham), the cerebral ischemia model group (Is), the Rapamycin group (RAPA), and the 3-Methyladenine group (3-MA). RAPA and 3-MA were diluted in a vehicle solution that contained saline dissolved in 1% dimethyl sulfoxide (DMSO). The rats were intraperitoneally injected with RAPA (4 mg/kg body weight) [39,40] and 3-MA (5 mg/kg body weight) [41,42] at 12 h prior to cerebral ischemia. An equivalent volume of vehicle solution was administered to Sham and Is groups.

### 2.3. Cell Culture and OGD Exposure

The PC12 rat adrenal pheochromocytoma cell line was obtained from Boster Biological Technology Co., Ltd. (Wuhan, China). Cells were cultured in Dulbecco’s Modified Eagle Medium (DMEM), supplemented with 10% fetal bovine serum (FBS) and 100 U/mL penicillin, and were maintained at a 37 °C incubator temperature with 5% CO_2_ with 95% humidity. The PC12 cells were separated into three groups: control group (CON), OGD group (OGD), and the muscimol group (Mus). OGD treatments were performed in a hypoxia chamber (Jinfeng Science and Technology Co., Ltd., Beijing, China) filled with 94% N_2_, 1% O_2_, and 5% CO_2_. During OGD treatment, cells were cultured in glucose-free DMEM for 2 h, and then they were moved to a normal medium in a regular incubator for 24 h [36,43]. Prior to OGD model induction, PC12 cells were pretreated with muscimol (1 umol/L).

### 2.4. Cell Viability Assay

We evaluated the effects of muscimol on the viability of PC12 cells using the Cell Counting Kit-8 assay (CCK-8, cat. # JD226, Beijing Jude Antai Technology Co., Ltd., Beijing, China). The cells were seeded into 96-well plates, and then 10 μL of CCK-8 reagent was added to PC12 cells. These were then incubated at 37 °C for 1 h. The optical density (OD) value was measured using a microplate reader at 450 nm.

### 2.5. Open-Field Test

After 24 h of operation, the behavior of rats was tested using an open-field test, as previously described [44]. Briefly, each rat was placed into the square open-field apparatus (100 cm × 100 cm × 50 cm), which was divided into 25 squares. The center area was defined as the central 9 squares of the open field. Every rat was observed for 5 min, and moving traces of rat in the test were recorded via a camera using ANY-maze video tracking system (Stoelting Co., Wood Dale, IL, USA). The total distance and duration in the center zone were quantified using this video tracking system.

### 2.6. Nissl Staining

After 24 h of cerebral ischemia (the behavioral tests were completed), the animals were anesthetized and perfused to collect brain tissue, which was fixed with 4% paraformaldehyde (PFA), embedded in an embedding medium, and sectioned to obtain 30 μm thick coronal slices. Next, the slices were dehydrated using gradient alcohol and rendered transparent using xylene. Finally, the slices were dyed with Nissl dye and then sealed using neutralresinsize.

### 2.7. Immunohistochemistry

The brain slices were rinsed in PBS and incubated in a 2% hydrogen peroxide solution for 1 h. They were then washed with 5 mM phosphate-buffered saline containing 0.3% Triton X-100 (PBS-X) three times. Next, the brain slices were incubated overnight with anti-NeuN antibody (Cat #MAB377, 1:500, Millipore, Billerica, MA, USA). After rinsing with PBS-X, the slices were incubated for 4 h with anti-mouse IgG (Cat 715-065-151, 1:100; Jackson, West Grove, PA, USA) and then for 1 h with ABC (1:50; Vector, Torrance, CA, USA). Finally, the slices were incubated with DAB (Sigma, Burlington, MA, USA) and 0.0001% H_2_O_2_ in 50 mM Tris-HCl for 5 min.

### 2.8. ELISA Measurement

The levels of GABA_A_R and GABA in brain tissue and cells were evaluated using ELISA assay kits. In addition, the concentrations of inflammatory factors (IL-1β, TGF-β and IL-10) were also assessed using ELISA assay kits. All procedures were conducted according to the manufacturer’s specifications.

### 2.9. Western Blot Analysis

The total protein was extracted using a total protein extraction kit (Biochain, Hayward, CA, USA) and quantified using a bicinchoninic acid (BCA) protein assay kit (78510, Pierce, Rockford, IL, USA). After sodium dodecyl sulfate polyacrylamide gel electrophoresis (SDS-PAGE), the gel was transferred to a polyvinyl difluoridine (PVDF) membrane (Millipore, Billerica, MA, USA) and immunoblotted using primary and secondary antibodies against GABA_A_R (1:1000; Cat No. AB33299, Abcam, Cambridge, MA, USA), Beclin-1 (1:1000; Cat No. 3738S, CST, Danvers, MA, USA), MAPLC3β (1:1000; Cat No. 12741T, CST, Danvers, MA, USA), Cathepsin B (1:1000; Cat No. 31718S, CST, Danvers, MA, USA), Phospho-AMP-activated protein kinase (p-AMPK) (1:1000; Cat No. 2535T, CST, Danvers, MA, USA), Phospho-mammalian target of rapamycin (p-mTOR) (1:1000; Cat No. 2983T, CST, Danvers, MA, USA), Bax (1:1000; Cat No. 2772S, CST, Danvers, MA, USA), Bcl-2 (1:1000; Cat No. 3498S, CST, Danvers, MA, USA), and β-actin (1:5000; Cat No. 50201, Kemei Borui Science and Technology Co., Ltd., Beijing, China). Then, membranes were incubated for 1 h at 37 °C with a horseradish peroxidase (HRP)-conjugated secondary antibody. Finally, an ECL chemiluminescence instrument (5200, Tanon Science & Technology Co., Ltd., Shanghai, China) was used to develop the membrane.

### 2.10. Immunofluorescence Assays

After appropriate treatment, fixed PC12 cells were permeabilized with 1% Triton X-100 at 37 °C for 2 h. They were then blocked with 5% donkey serum at 37 °C for 1 h and incubated with primary antibody at 4 °C for 48 h. Next, we performed double labeling with GABA_A_R and MAPLC3β. After primary antibody incubation, the samples were incubated with biotinylated secondary antibody at 4 °C for 24 h. Next, the samples were then incubated with the fluorescent antibody at 4 °C for 24 h. The samples were double-labeled with GABA_A_R and MAPLC3β. Cell nuclei were stained with DAPI (0100-20; SouthernBiotech, Birmingham, AL, USA) for 5 min at room temperature. The staining procedures for negative control sections were identical, except for the fact that the primary antibody was replaced with PBS. Fixed PC12 cells were imaged using an Eclipse Ti-U microscope (Nikon, Tokyo, Japan).

### 2.11. Measurement of Oxidative Stress Indexes

The degree of lipid peroxidation in the brain tissue of rats was determined based on malondialdehyde (MDA) level. The antioxidant capacity was evaluated in relation to the superoxide dismutase (SOD) activity and glutathione system (GSH) content. In brief, the brain tissues were washed thoroughly with PBS and then homogenized and centrifuged at 5000 rpm for 10 min to collect the supernatant, which was measured using the ROS detection kit (E004, NJJCBio Inc., Nanjing, China), MDA detection kit (A003-2, NJJCBio Inc., Nanjing, China), SOD detection kit (A001-1, NJJCBio Inc., Nanjing, China), and GSH detection kit (A006-1, NJJCBio Inc., Nanjing, China). All procedures were conducted according to the manufacturer’s instructions.

### 2.12. Transmission Electron Microscopy

The brains were quickly removed, and several pieces of 1 mm^3^ peri-ischemic cortex tissues were dissected and fixed in 2.5% glutaraldehyde in 0.2 mol/L PBS for at least 2 h at 4 °C. The samples were dehydrated with a graded series of ethanol and acetone and then embedded and cured in epoxy resin. Ultrathin sections were cut using an ultramicrotome and then double-stained with uranyl acetate and lead citrate. The ultrastructure of mitochondrial morphology was observed using a TEM-1400 Plus electron microscope (JEOL JEM-1400 Plus, Tokyo, Japan).

### 2.13. Measurement of NO and NOS Contents

NO production in the brain tissue was assessed utilizing the nitrate reductase method with an NO assay kit (A012, Njjcbio, Inc., Nanjing, China) in accordance with the manufacturer’s guidelines, and the content was given in µmol/g protein. The enzymatic activities of NO, total NOS, constitutive NOS (cNOS), and inducible NOS (iNOS) were determined using an NOS assay kit (A014-1, Njjcbio, Inc., Nanjing, China) following the manufacturer’s protocols, and the concentration was shown as U/mg protein.

### 2.14. Statistical Analysis

Data are presented as mean ± standard error mean (SEM). Statistical analyses were performed using one-way-ANOVA, followed by Tukey’s post hoc test performed with GraphPad Prism 8 (GraphPad Software, La Jolla, CA, USA). At least six rats in each group were required for statistical significance, and three independent experiments were conducted to confirm the results. Statistical significance values were defined as follows: * *p* < 0.05 or ^#^ *p* < 0.05 was considered significant.

## 3. Results

### 3.1. The Activation of GABA_A_R Regulated GABAergic Signaling and Attenuated Neuronal Damage Caused by Cerebral Ischemia

**Figure 1:**
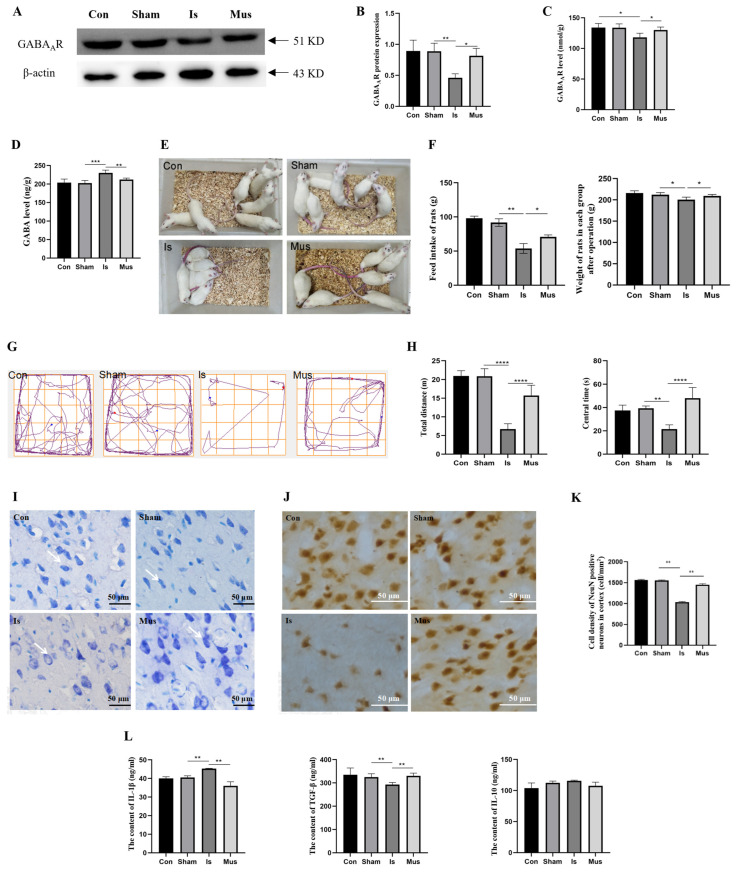
The activation of GABA_A_R regulated GABAergic signaling and attenuated neuronal damage caused by cerebral ischemia in rats. (**A**,**B**) Western blot analysis of GABA_A_R protein and relative expression levels in brain tissue. (**C**) The ELISA assay detected the concentration of GABA_A_R in brain tissue. (**D**) The content of GABA in brain tissue, as evaluated by ELISA assay. (**E**) Rat behavior and physical appearance. (**F**) Weight loss and feed intake. (**G**) Representative moving traces in the open-field test. (**H**) Measurement of total distance (left) and center time (right). (**I**) Changes in cortical neurons via Nissl staining (40×). White arrows indicate Nissl-stained neurons. (**J**,**K**) Distribution of NeuN-positive neurons in the cerebral cortex, as depicted via immunohistochemistry staining, and cell density of NeuN-positive neurons. (**L**) The content of IL-1β, TGF-β and IL-10 in brain tissue. Data are expressed as mean ± SEM; *n* = 6. * *p* < 0. 05, ** *p* < 0.01, *** *p* < 0.001, and **** *p* < 0.0001. GABA, γ-aminobutyric acid; GABA_A_R, GABA type A receptor.

Cortical GABAergic signaling via the activation of GABA_A_R exerts protective effects against cerebral ischemic injury. In order to investigate the role of GABA_A_R in cerebral ischemia, we conducted Western blot analysis, enabling the assessment of the protein level of GABA_A_R. Our results revealed that there was a significant decrease in GABA_A_R protein expression in the Is group compared to the Sham group. However, this downregulation was reversed when rats were pretreated with muscimol (Figure 1A,B). Moreover, there was no significant difference between the control and the Sham groups. Similar results were consistently obtained via the ELISA assay (Figure 1C). We further investigated the change in GABAergic signaling during cerebral ischemia and found that the contents of GABA were increased after cerebral ischemia compared with the Sham group, which was recovered by muscimol (Figure 1D). These findings suggest that the activation of GABA_A_R might protect against cerebral ischemic damage and regulate GABAergic signaling during cerebral ischemia.

We next explored the protective effects of muscimol on cerebral ischemia by performing assessments of behavioral performance and neuronal damage. Cerebral ischemia contributed to a deficiency in exploratory behavior, increased vigilance, and the appearance of vertical fur and dry hair in rats, but these effects were significantly reversed via pretreatment with muscimol (Figure 1E). In addition, the rats of the Is group showed obvious weight loss and decreased feed intake. However, the detrimental effects were markedly reduced via pretreatment with muscimol (Figure 1F). Additionally, the rats in the Mus group moved a longer total distance and stayed in the center for a longer period of time compared with the Is group, which demonstrated that muscimol pretreatment normalized the anxiety behaviors induced by cerebral ischemia (Figure 1G,H).

Consistent with our previous study [36], Nissl staining results revealed that, in the control and Sham groups, the dark-blue-stained granular Nissl bodies showed normal histomorphology and regular arrangement. In the Is group, neurons were damaged to different degrees, with a large loss of Nissl bodies, abnormal morphological structure, and disorganized and loosely arranged cells. In contrast with the Is group, pretreatment with muscimol markedly attenuated the neuronal damage caused by cerebral ischemia, helping to maintain the number of neurons and improve the integrity of the morphological structure (Figure 1I). In addition, there was a significant reduction in the number of NeuN-positive cells in the Is group, which might be effectively counteracted by muscimol (Figure 1J,K). Neuronal damage elicits a complex cascade of events involving immune cell activation, ultimately leading to the occurrence of inflammation [45]. We further evaluated the effect of activated GABA_A_R on inflammatory cytokines. The results showed that cerebral stroke led to effectively upregulated IL-1β levels but significantly decreased TGF-β content, which was reversed by muscimol. Nevertheless, there was no remarkable difference in the level of IL-10 among groups (Figure 1L). The data above emphasize that GABA_A_R activation relieves neuronal damage by diminishing neuroinflammation in cerebral ischemia.

### 3.2. The Activation of GABA_A_R Alleviated Cerebral Ischemia-Induced Oxidative Stress via Inhibiting the NO/NOS Pathway

**Figure 2:**
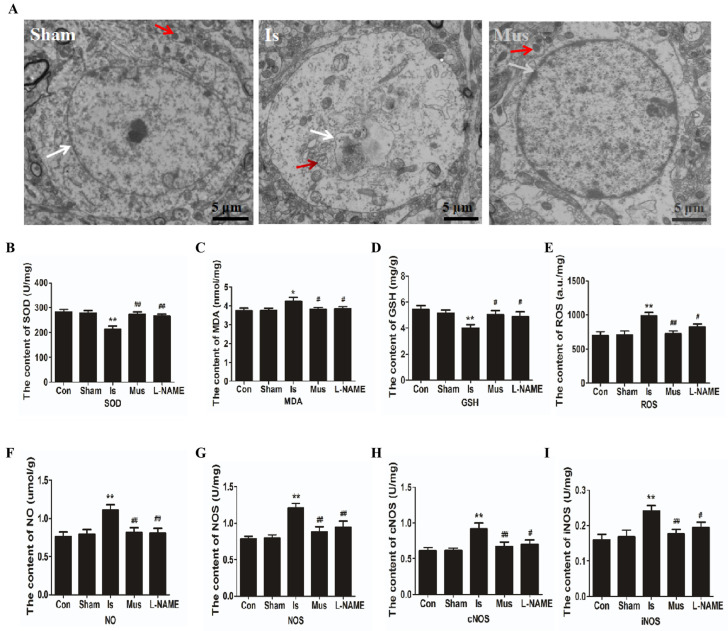
The activation of GABA_A_R alleviated cerebral ischemia-induced oxidative stress via inhibiting the NO/NOS pathway in rats. (**A**) Representative transmission electron microscopy (TEM) images of the mitochondrial structure of neurons in the cerebral cortex. Mitochondria are indicated by red arrows and nuclear membrane is indicated by white arrows. Scale bar = 5 μm. (**B**–**I**) The contents of SOD (**B**), MDA (**C**), GSH (**D**), ROS (**E**), NO (**F**), NOS (**G**), cNOS (**H**), and iNOS (**I**) in brain tissue of each group during cerebral ischemia. Data are expressed as mean ± SEM; *n* = 6. * *p* < 0.05 and ** *p* < 0.01 vs. the Sham group. ^#^ *p* < 0.05 and ^##^ *p* < 0.01 vs. the Is group. MDA, malondialdehyde; SOD, superoxide dismutase; GSH, glutathione system; ROS, reactive oxygen species; NO, nitric oxide; NOS, nitric oxide synthase; cNOS, constitutive NOS; iNOS, inducible NOS.

Oxidative stress is a key component of cerebral ischemic injury. One outcome of oxidative stress in cells is damage to mitochondrial membranes, often accompanied by mitochondrial dysfunction [46]. Therefore, we observed the mitochondrial structure of neurons in the cerebral cortex under different treatment conditions in rats using TEM. As shown in Figure 2A, our results revealed the fracturing of mitochondrial cristae. The disappearance of membranes and vacuoles was apparent in the Is group following ischemic stroke, while muscimol pretreatment alleviated mitochondrial damages. These results imply the protective effect of activated GABA_A_R against mitochondrial dysfunction induced by cerebral ischemia. Several biochemical analyses of brain tissue in rats were further performed to investigate the impacts of activated GABA_A_R on oxidative stress after cerebral ischemia. As shown in Figure 2B–D, no significant difference was found in the content of SOD, MDA, and GSH between the control group and the Sham group. SOD activity and GSH level increased remarkably, but the content of MDA was reduced in comparison with the control group. This was effectively reversed via treatment with L-nitroarginine methyl ester (L-NAME), a nonselective NOS inhibitor, suggesting that NOS generation contributed to reducing oxidative damage. Conversely, the Mus group revealed an improvement in SOD activity and GSH level, as well as a decrease in MDA production to a level similar to that of the control group. In addition, there was an obvious elevation of ROS level in the brain tissue after ischemic stroke compared with the control group. In contrast, the ROS level of the Is group markedly reduced when rats were pretreated with muscimol (Figure 2E). These data indicate that muscimol enhances the antioxidant defense system, reducing oxidative damage during ischemia.

To elucidate the potential antioxidative mechanism of GABA_A_R activation, we further examined the NO/NOS signaling pathway in brain tissue. We found that, compared to the control group, no obvious changes were observed in the contents of NO/NOS/cNOS/iNOS of the Sham group. Interestingly, compared to the Sham group, the levels of NO, NOS, cNOS, and iNOS were significantly elevated in the Is group, which effectively reversed these changes via pretreatment with muscimol (Figure 2F–I). These findings suggest that GABA_A_R activation could mitigate the oxidative damage caused by stroke through modulating the NO/NOS signaling pathway.

### 3.3. Inhibition of Autophagy-Alleviated Cerebral Ischemic Injury in Rats

**Figure 3:**
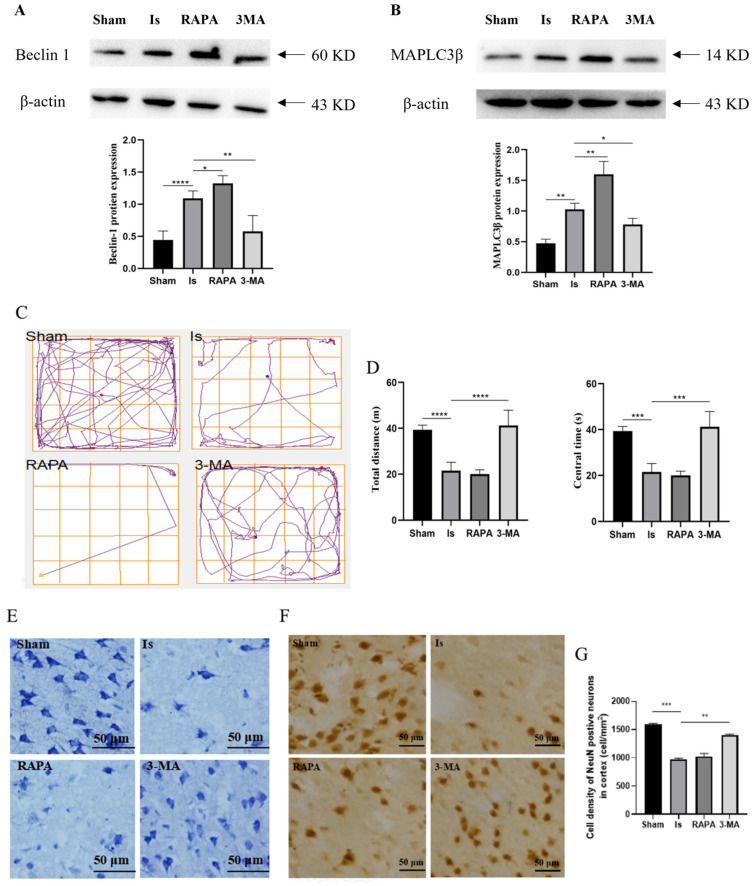
Inhibition of autophagy-alleviated cerebral ischemic injury in rats. (**A**,**B**) The expression of Beclin-1 and MAPLC3β in brain tissue, as determined via Western blotting and quantitative density data analyses. (**C**) Representative moving traces in the open-field test. (**D**) Measurement of total distance (left) and center time (right). (**E**) Cortical neurons as depicted by Nissl staining (40×). Scale bar = 50 μm. (**F**) The NeuN-positive cells in the cerebral cortex as assessed via immunohistochemistry staining. Scale bar = 50 μm. (**G**) Cell density of NeuN-positive neurons in the cerebral cortex. Data are expressed as mean ± SEM; *n* = 6. * *p* < 0. 05, ** *p* < 0.01, *** *p* < 0.001, and **** *p* < 0.0001.

The autophagic process is closely involved in the pathogenesis and progression of ischemic stroke, and the accumulation of oxidation products can lead to the induction of autophagy in cerebral ischemic injury. Therefore, we speculate that there may be alterations in autophagy during stroke. As shown in Figure 3A,B, the protein expression of Beclin-1 and MAPLC3β in the Is group was obviously higher compared to the Sham group. After treatment with RAPA, the Beclin-1 and MAPLC3β protein levels were augmented significantly above that of the Is group but were markedly decreased after 3-MA treatment.

To investigate the roles of autophagy in cerebral ischemia, the anxiety behavior of rats was observed 24 h after ischemic induction via an open-field test. The results showed that, compared with the Sham group, the total distance and central time of rats in the Is group were significantly decreased. However, compared with the Is group, the pretreatment of 3-MA significantly increased the total distance and central time. Conversely, rapamycin treatment had no effect on the Is group (Figure 3C,D). Nissl staining suggested that, in comparison with the Sham group, cerebral ischemia caused severe neuronal damage due to irregular cellular arrangement and the loss of massive Nissl bodies. The RAPA group saw similar results to the Is group, but the neuronal damage was improved in the 3-MA group compared with the Is group, including a reduction in the number of atrophic or necrotic neurons, a partial improvement in the morphological structural integrity, and an increase in the regular cell arrangement (Figure 3E). We further evaluated the injury degree of cortical neurons induced by ischemia, and the number of NeuN-positive cells in the cortex was calculated via immunohistochemistry. In the Is group, the NeuN-positive cell number was significantly lower than that in the Sham group. Compared with the Is group, the pretreatment of 3-MA markedly elevated the NeuN-positive cell, and no remarkable dissimilarity was detected in the RAPA group (Figure 3F,G). These results suggest that cerebral ischemia facilitated the process of autophagy, and 3-MA exerted protective effects against ischemia-caused brain insult by inhibiting autophagy.

### 3.4. The Activation of GABA_A_R Inhibited Ischemia-Induced Autophagy through the AMPK/mTOR Signaling Pathway

**Figure 4:**
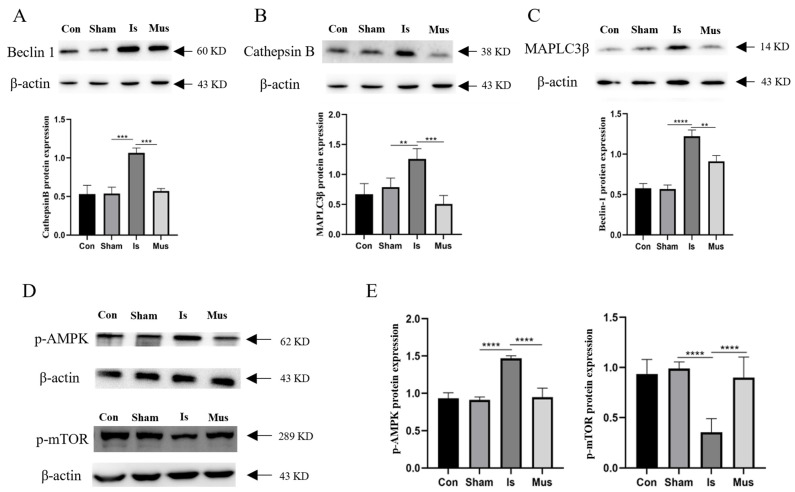
The activation of GABA_A_R suppressed autophagy through the AMPK/mTOR signaling pathway in rats of cerebral ischemia. (**A**) The expression of Beclin-1 protein in brain tissue, as determined via Western blotting and quantitative density data analyses. (**B**) Representative Western blot images and quantification of Cathepsin B protein level in brain tissue. (**C**) Expression of MAPLC3β protein in brain tissue, as examined by Western blot and relative expression levels. (**D**,**E**). The protein expression of p-AMPK and p-mTOR and quantification of relative expression in brain tissue. Data are expressed as mean ± SEM; *n* = 6. ** *p* < 0.01, *** *p* < 0.001, and **** *p* < 0.0001.

Considering the above results demonstrating that the inhibition of autophagy could improve cerebral ischemia, we investigated whether GABA_A_R activation regulates autophagy induced by ischemic stroke and, thus, exerts neuroprotective effects. Compared to the control group, the Sham group showed few obvious alterations in autophagy-related proteins. In comparison with the Sham group, cerebral ischemia caused a notable upregulation in the expression of Beclin-1, Cathepsin B, and MAPLC3β. However, pretreatment with muscimol significantly decreased these protein expression levels relative to the Is group (Figure 4A–C). The AMPK/mTOR signaling pathway is known to intricately regulate autophagy, and we further hypothesized that muscimol might modulate autophagy through the AMPK/mTOR signaling pathway. Therefore, we measured the expression of proteins associated with the AMPK/mTOR signaling pathway. Interestingly, compared to the control and Sham groups, the p-AMPK protein expression in the Is group was significantly increased, but the protein level of p-mTOR was reduced. In contrast, the Mus group displayed a decreased p-AMPK protein level and augmented p-mTOR expression relative to the Is group (Figure 4D,E). Collectively, these data underscore that GABA_A_R activation may confer neuroprotection during cerebral ischemia by inhibiting autophagy via the AMPK/mTOR signaling pathway.

**Figure 5:**
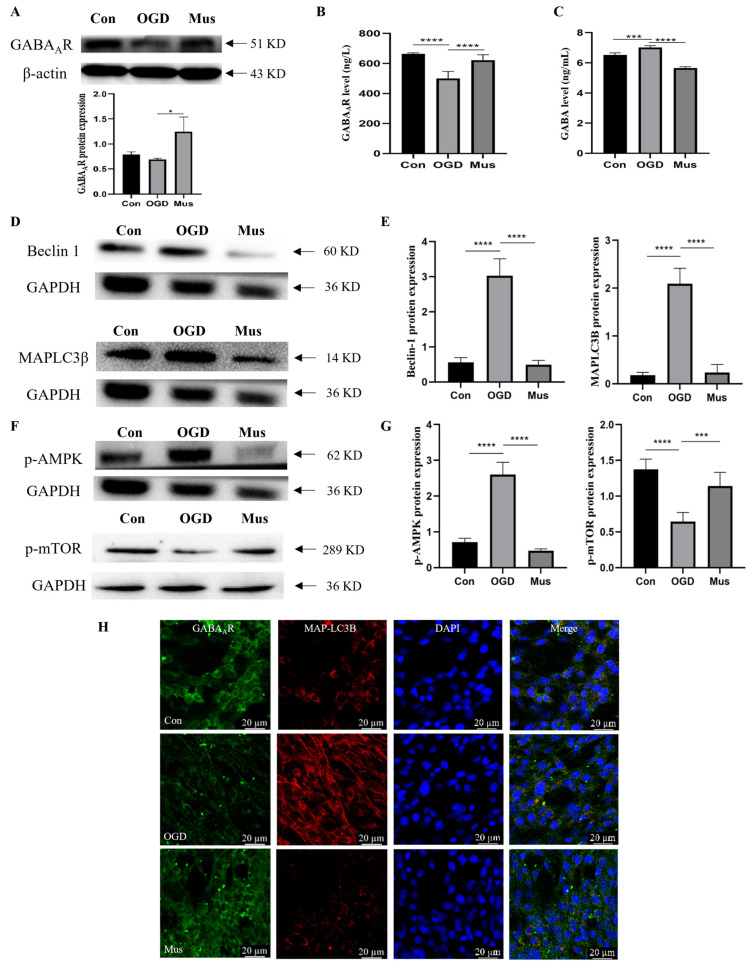
The activation of GABA_A_R suppressed autophagy through the AMPK/mTOR signaling pathway in OGD-exposed PC12 cells. (**A**) Representative images of Western blot and quantification of GABA_A_R protein level in PC12 cells. (**B**) ELISA assay tested the contents of GABA_A_R in PC12 cells. (**C**) The level of GABA in PC12 cells, as evaluated via ELISA assay. (**D**,**E**) Representative Western blot images and quantification of Beclin-1 and MAPLC3β protein levels in PC12 cells. (**F**,**G**) Western blot analysis of p-AMPK and p-mTOR protein level, as well as their relative expression in PC12 cells. (**H**) Double immunofluorescence of GABA_A_R (green) and MAPLC3β (autophagy marker, red) in PC12 cells in Con, OGD, and Mus groups. Images were captured via confocal microscopy. Scale bar = 20 μm. Data are expressed as mean ± SEM; *n* = 6. * *p* < 0.05, *** *p* < 0.001 and **** *p* < 0.0001.

Then, following exposure to OGD to mimic cerebral ischemia, we applied PC12 cells in vitro to investigate the effect of GABA_A_R activation on ischemic injury. Western blot analysis was performed to assess changes in GABA_A_R expression. It was established that, similar to the in vivo results, GABA_A_R protein expression was significantly decreased in the OGD group compared to the control group. However, the protein level of GABA_A_R was effectively enhanced in the Mus group relative to the OGD group (Figure 5A). Consistent with the above results, the content of GABA_A_R was decreased in the Is group, which was reversed via muscimol addition (Figure 5B). In addition, OGD exposure augmented the contents of GABA in comparison with the Sham group, as recovered via muscimol (Figure 5C). The results above suggest that activated GABA_A_R regulates OGD-induced GABAergic signaling in PC12 cells.

Additionally, we investigated the impact of GABA_A_R activation on autophagy in OGD-exposed PC12 cells. Compared with the control group, the expression level of Beclin-1 and MAPLC3β was obviously increased in the OGD exposure group, whereas muscimol pretreatment effectively abolished this effect (Figure 5D,E). To further explore the regulatory mechanism of GABA_A_R activation on autophagy in PC12 cells after OGD exposure, Western blot analysis was used to assess the key molecules in AMPK/mTOR signaling. The results revealed that the p-mTOR protein level was significantly decreased, but that p-AMPK expression was upregulated following OGD exposure relative to the control group, which was effectively reversed via muscimol addition (Figure 5F,G). Double immunofluorescence results verified the colocalization of GABA_A_R and MAPLC3β in PC12 cells under different treatments. Additionally, we found that pretreatment with muscimol strengthened GABA_A_R expression and markedly suppressed OGD-stimulated MAPLC3β signal (Figure 5H). These data suggest that GABA_A_R activation attenuates OGD injury by dampening autophagy via AMPK-mTOR signaling pathways.

### 3.5. The Activation of GABA_A_R Inhibited Ischemia-Induced Apoptosis through Modulating the Bcl-2/Bax Signaling Pathway

**Figure 6:**
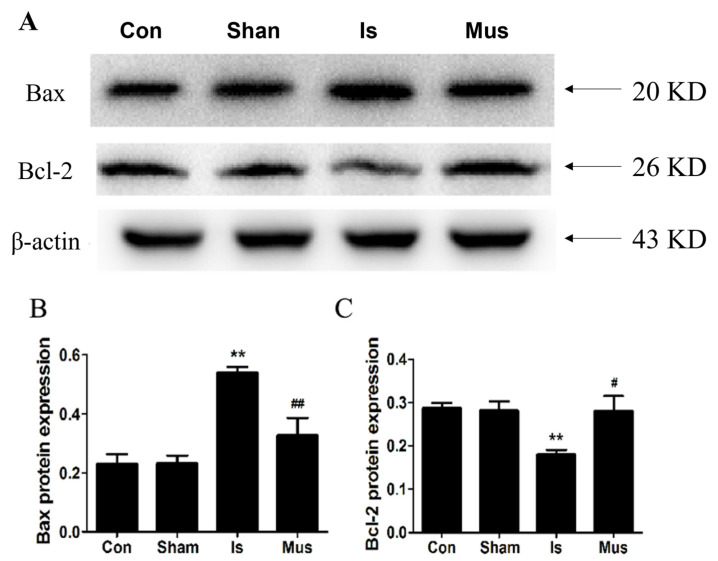
The activation of GABA_A_R suppressed apoptosis by regulating the Bcl-2/Bax signaling pathway in rats of cerebral ischemia. (**A**) Protein expression of Bax and Bcl-2 in brain tissues by Western blot. (**B**,**C**) Image J analysis of Bax and Bcl-2 protein levels in each group. Data are expressed as mean ± SEM; *n* = 6. ** *p* < 0.01 vs. the Sham group. ^#^ *p* < 0.05 and ^##^ *p* < 0.01 vs. the Is group.

Autophagy has been shown to trigger apoptosis during cerebral ischemic injury [47], and a growing body of research suggests that apoptosis plays a critical role in neuronal death after ischemic injury. We verified whether GABA_A_R activation exerted an anti-apoptosis effect. The protein expressions of Bax and Bcl-2 were assessed, and no significant difference was found in Bax and Bcl-2 expression between the Sham and the control groups. Conversely, the level of anti-apoptotic Bcl-2 protein was remarkably downregulated, but the pro-apoptotic Bax protein level was effectively augmented in the Is group. By contrast, muscimol pretreatment markedly elevated Bcl-2 expression but reduced the Bax protein level (Figure 6A–C).

**Figure 7:**
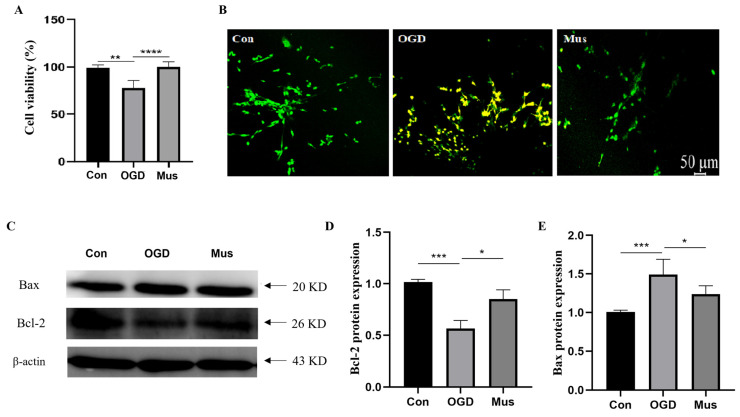
The activation of GABA_A_R suppressed apoptosis by regulating the Bcl-2/Bax signaling pathway in OGD-induced PC12 cells. (**A**) Cell viability of PC12 cells as assessed via CCK8 assay. (**B**) The apoptosis of PC12 cells via AO/EB staining. (**C**) Representative expression of Bcl-2 and Bax proteins in PC12 cells as identified via Western blotting. (**D**,**E**) Quantification of Bax and Bcl-2 protein levels in PC12 cells. Data are expressed as mean ± SEM; *n* = 6. * *p* < 0.05, ** *p* < 0.01, *** *p* < 0.001, and **** *p* < 0.0001.

To investigate whether GABA_A_R activation prevents PC12 cells against OGD damage, we assayed the cell viability of PC12 cells exposed to OGD. The CCK-8 assay showed that OGD markedly impaired the viability of PC12 cells, whereas pretreatment with muscimol was sufficient to the task of promoting cell proliferation compared to the OGD group (Figure 7A). The detection of apoptosis through AO/EB staining showed that no apparent apoptosis was detected in the control group. Along with this, early apoptotic cells with yellow-green fluorescence were observed in the OGD group. However, the yellow-green fluorescence intensity was reduced in the Mus group compared to the OGD group, exerting an anti-apoptotic effect (Figure 7B). In addition, Western blot was conducted to test whether activated GABA_A_R could affect apoptosis-related protein expression under OGD exposure. In comparison with the control group, OGD remarkably resulted in a reduced Bcl-2 level and upregulated Bax expression, whereas muscimol markedly promoted Bcl-2 protein expression but repressed Bax protein levels (Figure 7C,E). These results highlight that the activation of GABA_A_R could protect against OGD-induced cell apoptosis.

## 4. Discussion

In the present study, we investigated the neuroprotective effects of GABA_A_R activation and its potential mechanisms during cerebral ischemia. Our data demonstrate that the activation of GABA_A_R alleviated clinical symptoms and mitigated the neuronal damage induced by cerebral ischemia via the modulation of oxidative stress and inhibition of autophagy and apoptosis pathways. Specifically, GABA_A_R activation suppressed autophagy by modulating the AMPK-mTOR signaling pathway. Moreover, the regulation of oxidative stress by activated GABA_A_R was closely related to the NO/NOS pathway.

Ischemic stroke causes high mortality and disability rates worldwide, which have enormous social and economic impacts [48]. Recent studies have illustrated that stroke and COVID-19 share risk factors. Indeed, ischemic stroke has become the most common cerebrovascular complication in patients with COVID-19 [49]. The concentration of glutamate and aspartate in the brain of rats with cerebral ischemia is greatly increased, and glutamate-mediated excitotoxicity is the principal cause of ischemic neuronal death [50,51]. GABA is an important inhibitory neurotransmitter, playing an important role in regulating excitatory and inhibitory balance in the brain [52]. There was evidence suggested that muscimol has been used to reduce ischemic injury in clinical and animal trials, displaying the powerful ability to activate GABA_A_R signaling [53]. Consistent with the results of previous studies, we found that pretreatment with muscimol enhanced GABA_A_R expression. There is already ample evidence that ischemic episodes lead to large and dramatic increases in GABA concentration in the brain alongside the simultaneous inhibition of GABA synthesis and release, resulting in a decrease in GABA function [28], which is consistent with what we observed. Remarkably, muscimol significantly decreased the contents of GABA in the Is rats or OGD-induced cells, a possibility that we attributed to the restoration of endogenous GABA synthesis and intrasynaptic signaling after the activation of GABA_A_R, which reversed the short-lived compensatory elevation of GABA that occurs during ischemia. We further confirmed that muscimol mitigated behavioral performance and cortical neuronal damage during cerebral ischemia. Consistent with the results of our work, muscimol has been reported to improve learning/memory deficits in a streptozocin-induced Alzheimer’s-disease rat model and prevent neuronal injury [54]. Neuronal injury was accompanied by neuroinflammation, which plays a crucial role in the pathophysiology of cerebral ischemia [45]. Our study revealed that ischemic stroke contributed to the upregulated pro-inflammatory IL-1β level but decreased anti-inflammatory TFG-β content, which was consistent with previous studies [55]. However, GABA_A_R activation effectively reversed these changes, which suggested that the activation of GABA_A_R attenuated the neurological damage and inflammatory response induced by cerebral ischemia.

Oxidative stress, characterized by an imbalance between the production and removal of ROS, is a pivotal factor in ischemic brain injury [56]. The deleterious effects of excess ROS and RNS include organelle swelling and necrosis, lipid peroxidation, as well as induction of autophagy and apoptosis [57]. It has been previously reported that reactive oxygen species may compromise GABA(A)-mediated neuronal inhibition, and that a reduction in GABA(A)-gated Cl^−^ channel function during periods of oxidative stress may contribute to the development of neuronal damage [58,59]. Therefore, enhanced antioxidant activity plays a vital role in attenuating ischemic insult. We found that there was a reduction in ROS level when rats were treated using muscimol. Furthermore, SOD and GSH are involved in antioxidant processes, and the MDA level reflects the degree of oxidative stress [60]. In the current study, the levels of SOD and GSH were significantly increased by muscimol pretreatment, while the concentration of MDA was reduced. One outcome of oxidative stress is that damage is inflicted to mitochondrial membranes. As expected, muscimol notably inhibited the mitochondrial damage induced by cerebral ischemia. It has been reported that NOS and NO production are related to the aggravation of oxidative stress, in which NO is mainly generated via nNOS in the early stages of cerebral ischemia, while the late phase sees NO primarily induced by iNOS [9,61]. Our results agree with previous research: L-NAME obviously restored oxidative damage caused by cerebral ischemia by suppressing NOS expression, and GABA_A_R activation inhibited oxidative stress by impeding NO/NOS pathway during the cerebral ischemia.

Oxidative stress and autophagy interact, and the accumulation of oxidation products can lead to an autophagic induction in cerebral ischemic injury [62]. Additionally, autophagy acts as a major sensor of redox signaling during ischemic stroke. Numerous studies have shown that neuronal autophagy after ischemic stroke is among the most important mechanisms of cerebral ischemia injury [13]. Moderate autophagy may contribute to the degradation of aggregated proteins, while impaired or excessive autophagy can ultimately lead to cell death, and the dual role of autophagy has garnered significant attention [63,64]. However, its role in ischemic stroke remains controversial. Beclin-1, as the representative marker of autophagy, plays a crucial role in autophagosome formation [65,66]. MAPLC3β is another vital autophagy marker that mediates the fusion of autophagosomes with lysosomes [67]. In our study, based on an established rat model of cerebral ischemia, we used rapamycin (an autophagy agonist) and 3-MA (an autophagy inhibitor), respectively. Our results showed that 3-MA reduced the levels of Beclin-1 and MAPLC3β in cerebral ischemia and alleviated the behavioral abnormalities and neuronal damage caused by cerebral ischemia. It is worth noting that, compared with the Is group, RAPA did not significantly change, which was probably due to the excessive autophagy induced by ischemia. Our data suggest that autophagy activation was significantly increased during cerebral ischemia and that the inhibition of autophagy could be neuroprotective in cerebral ischemic rats. In line with previous findings, the intracerebroventricular injection of 3-MA after ischemia was found to be capable of significantly inhibiting neuronal autophagy and reducing infarct volume in rats exposed to transient focal cerebral ischemia [17,68].

Moreover, considering the adverse role of overactive autophagy in the process of stroke, we found that muscimol decreased the expression levels of Beclin-1, Cathepsin B, and MAPLC3β in cerebral ischemic rats, which suggested that muscimol inhibited autophagy induced by cerebral ischemia. The results in vitro also confirmed that muscimol obviously suppressed autophagy by reducing Beclin-1 and MAPLC3β expression in OGD-exposed PC12 cells. Moreover, AMPK is a key regulator of autophagy in response to cellular energy depletion, and its downstream target mTOR negatively modulates autophagy [69]. In our work, muscimol obviously diminished p-AMPK protein expression but increased p-mTOR levels in both the Is rats and OGD-induced cells. We could confirm that the activation of GABA_A_R reduced cerebral ischemia-induced autophagy via the AMPK-mTOR signaling pathway.

There has been research seeking to elucidate how both oxidative stress and autophagy might lead to apoptosis. Bcl-2 is an anti-apoptotic protein that stabilizes mitochondrial membranes, while Bax is a pro-apoptotic protein that increases mitochondrial membrane permeability [70]. In our present observations, we found that muscimol could suppress apoptosis in rats of cerebral ischemia by reducing the ratio of Bax/Bcl-2, which was also illustrated in OGD-induced PC12 cells. Considering that an increase in NO production could reduce mitochondrial membrane potential and promote cytochrome C to migrate from the mitochondria to the cytoplasm, leading to apoptosis [71,72], we could infer that GABA_A_R activation might repress apoptosis via the downregulation of NO/NOS-mediated oxidative stress. In one such study, AMPK-mediated autophagy enhanced oxidative stress and induced apoptosis in ischemic stroke models [73]. Thus, our work provides some clues that the activation of GABA_A_R relieves cerebral ischemia via regulating the crosstalk between oxidative stress, autophagy, and apoptosis pathway.

We would like to highlight the limitations of the present study. We found that the agonist of GABA_A_R, muscimol, attenuated cerebral ischemic injury by modulating oxidative stress, autophagy, and apoptosis. However, to fully elucidate the GABA_A_R-mediated effects, the role of GABA_A_R inhibitors during cerebral ischemia should also be considered. Moreover, the inter-regulatory effects among oxidative stress, autophagy, and apoptosis after GABA_A_R activation require further exploration in future studies. Moreover, in our future research, we will also isolate primary neurons from the brain to better mimic the in vivo environment and further fully elucidate the neuroprotective effects of GABA_A_R against cerebral ischemia. Additionally, muscimol is not only a GABA_A_R agonist but also a sedative drug, which might interfere with brain metabolism and, thus, have beneficial effects against cerebral ischemic injury. Therefore, in the future, we should use more selective GABA_A_R agonists or apply gene editing techniques to eliminate the effects of other interfering factors.

## 5. Conclusions

In summary, the present study indicates that muscimol, a GABA_A_R agonist, modulates GABAergic signaling, improves anxiety-like behaviors, and attenuates neuronal damage in rats with cerebral ischemia. The activation of GABA_A_R exerts neuroprotective effects through improving oxidative stress and inhibiting autophagy and apoptosis. Furthermore, GABA_A_R activation inhibits autophagy by regulating the AMPK-mTOR pathway, thereby attenuating neuronal damage induced by cerebral ischemia in rats.

## Data Availability

All figures and data used to support this study are included within this article.
